# Effects of early-life conditions on innate immune function in adult zebra finches

**DOI:** 10.1242/jeb.242158

**Published:** 2021-06-04

**Authors:** Merijn M. G. Driessen, Maaike A. Versteegh, Yoran H. Gerritsma, B. Irene Tieleman, Ido Pen, Simon Verhulst

**Affiliations:** Groningen Institute for Evolutionary Life Sciences, University of Groningen, Nijenborgh 7, 9747 AG, Groningen, The Netherlands

**Keywords:** Innate immunity, Development, Brood size manipulation, Meso-population, *Taenopygia guttata*

## Abstract

Early life conditions can affect individuals for life, with harsh developmental conditions resulting in lower fitness, but the underlying mechanisms are not well understood. We hypothesized that immune function may be part of the underlying mechanism, when harsh developmental conditions result in less effective immune function. We tested this hypothesis by comparing innate immune function between zebra finches (*Taeniopygia guttata*) in adulthood (*n*=230; age 108–749 days) that were reared in either small or large broods. We used this experimental background to follow up our earlier finding that finches reared in large broods have a shorter lifespan. To render a broad overview of innate immune function, we used an array of six measures: bacterial killing capacity, hemagglutination, hemolysis, haptoglobin, nitric oxide and ovotransferrin. We found no convincing evidence for effects of natal brood size on any of the six measures of innate immune function. This raised the question whether the origin of variation in immune function was genetic, and we therefore estimated heritabilities using animal models. However, we found heritability estimates to be low (range 0.04–0.11) for all measured immune variables, suggesting variation in innate immune function can largely be attributed to environmental effects independent of early-life conditions as modified by natal brood size.

## Abstract (precis)

**Summary:** Developmental hardship has many long-term implications, but its effects on adult immune function are unknown. We found no effects of a developmental manipulation on innate immune function during adulthood in zebra finches.

## INTRODUCTION

The early developmental period is a sensitive life stage, and early-life conditions can have strong effects on fitness (Briga et al., 2017; Reid et al., 2003; Saino et al., 2018; Van De Pol et al., 2006). Presumably, such effects are mediated through long-term phenotypic effects of early-life conditions, shaping subsequent behavior, physiology and morphology. For example, manipulation of natal brood size, which typically affects growth, has long-term phenotypic effects on physiology (e.g. Briga et al., 2016; Jimeno et al., 2019; Naguib et al., 2004; Saino et al., 2018; Tschirren et al., 2009; Verhulst et al., 2006), sexual ornamentation (Gustafsson et al., 1995) and telomere length (Boonekamp et al., 2014). In captive zebra finches, growing up in large broods has been shown to cause a shortening of adult lifespan (Briga et al., 2017; De Kogel, 1997), but the mechanisms causing this effect remain to be elucidated.

The immune system is a potential mediator of fitness variation (e.g. Roast et al., 2020), and may thus explain long-term effects of developmental conditions on lifespan. Previous studies have shown that developmental hardship affects immune system components measured during the early developmental stages (Bourgeon and Guindre-parker, 2011; Moreno-Rueda, 2010; Moreno-Rueda and Redondo, 2011; Naguib et al., 2004; Saino et al., 1997), possibly owing to a trade-off between growth and immune function (Van Der Most et al., 2011). However, the long-term effects of developmental hardship on immune function are less well studied, and studies show mixed results usually based on a small number of immune markers. Low food availability during development led to a higher inflammatory immune response to phytohemagglutinin (PHA) in lizards (Mugabo et al., 2010) and male song sparrows (*Melospiza melodia*; Schmidt et al., 2015), but had no effect on *Candida albicans* killing in zebra finches (Kriengwatana et al., 2013), or on *C. albicans* killing or *Escherichia coli* killing, lysis and natural antibody levels in song sparrows (Schmidt et al., 2015). In the zebra finches, an effect on *E. coli* killing was observed dependent on later food availability (Kriengwatana et al., 2013). Brood size manipulations also yielded mixed results, with large broods leading to a higher PHA response in domestic, but not in wild-caught young adult zebra finches (Tschirren et al., 2009).

Effects of developmental manipulations on immune function arise against the background of the genetic makeup. Heritability estimates of immunity vary between immune indices and studies, and can be high in controlled conditions. For example, Clapperton et al. (2009), studying pigs in controlled specific pathogen-free conditions, found heritabilities ranging from 0.04 up to 0.82 for various white blood cell counts and acute phase proteins. However, innate immune system components have been found to have lower heritability, also in controlled conditions. For instance, heritability of haptoglobin in highly controlled settings ranged from 0.14 to 0.23 (Clapperton et al., 2009; Henryon et al., 2006). Heritability estimates are typically environment dependent, and little work has been done on heritability of immune function in ecological settings. From the few studies that have been done, we learned that heritabilities tend to be low. For example, Ardia and Rice (2006) reported PHA heritabilities close to zero in two out of three tree swallow (*Tachycineta bicolor*) populations.

In this study, we investigated the effects of manipulations of natal brood size on immune function during adulthood in zebra finches. In this model species, the effects of brood size manipulations have been well studied and are known to affect lifespan (Briga et al., 2017; De Kogel, 1997), and effects of developmental hardship on chick and juvenile immune function have previously been explored (Kriengwatana et al., 2013; Naguib et al., 2004; Tschirren et al., 2009). Here, we add by providing data on long-term effects of developmental hardship. We cross-fostered nestlings to create small and large broods, following Briga et al. (2017). During adulthood, the birds were housed in outdoor aviaries, exposed to natural weather conditions (Briga and Verhulst, 2015). We used six different measures of innate immune components, since the various parts of the immune system are often found to be poorly correlated and correlations vary between species (Matson et al., 2006). An overview of immune measures can be found in Table S1. We hypothesized that innate immune function is reduced in birds reared in large broods, given that this treatment shortened lifespan. Additionally, we investigated effects of sex and age on immune function, as well as any sex- and age-specific effects of the developmental treatment. Lastly, we estimated heritability of immune function using animal models and the known pedigree to put the potential effects of developmental conditions into perspective.

## MATERIALS AND METHODS

### Animals and treatment

Rearing conditions of the birds, 230 zebra finches [*Taeniopygia guttata* (Vieillot 1817)], were as described in Briga et al. (2017). In brief, parental birds were randomly mated and pairs were housed in cages (104×52×52 cm) with nesting material, cuttlefish bone, drinking water and commercial seed mixture *ad libitum*, with additional egg food supplementation up to hatching. When the chicks were 1–5 days old, they were weighed and randomly cross-fostered to other nests to create small (2–3 chicks) and large (5–7 chicks) broods, forming the benign and harsh treatments, respectively. These brood sizes are within the natural range (Zann, 1996). At age 35 days, the juveniles were separated from their foster parents and moved to indoor aviaries (153×70×110 cm) with up to 30 same-sex young and four adults, two of each sex, for sexual imprinting. Around age 100 days (range 90–120 days), individuals were moved to outdoor aviaries (310×210×150 cm), where they stayed until blood sampling at a later age (median: 263 days, range: 107–749 days). In the outdoor aviaries, birds were housed with up to 24 individuals per aviary, with *ad libitum* food and water.

The population of birds used in this study was built up over time for a large experiment, and were all sampled shortly before that experiment started. Hence there was a large range in age at sampling, which could bias our age estimates, but not our estimates of treatment or sex effects, because sampling was balanced with respect to these factors. With respect to selective disappearance, we note that we found no age effects (see below) and that the correlation between age and our experimental treatment was very low (point-biserial correlation, *r*_pb_=0.06), but we cannot exclude that age effects and selective disappearance cancelled each other out in our cross-sectional analysis. Longitudinal sampling will be required to unambiguously test for age effects (Peters et al., 2019), and this will be the subject of a later study.

All methods and experimental protocols were carried out under the approval of the Central Committee for Animal Experiments (Centrale Commissie Dierproeven) of The Netherlands, under license AVD1050020174344. All methods were carried out in accordance with these approved guidelines.

### Blood sampling and processing

Birds were sampled twice, with a 2-week interval, spread out over multiple weeks in either March or September (2018 or 2019), before they entered into a large follow-up study. Developmental treatment and sex were balanced with respect to season and we found no evidence for an interaction between age and season. To minimize any potential handling stress effects on immune parameters (Buehler et al., 2008a), we sampled birds within minutes after entering the aviary (median: 4 min, range 1–12 min between entering the aviary and the end of sampling), and no person had entered the aviary for at least 1 h prior to sampling.

We sterilized the wing by cotton swab with 70% ethanol prior to sampling. Blood (150 μl) was taken from the brachial vein by puncture and collected in heparinized capillaries, and immediately transferred into Eppendorf tubes on ice. Plasma was removed after centrifuging samples for 10 min at 1500 ***g*** and subsequently stored at −20°C until used in the immune assays.

### Immune tests

Prior to the assays, plasma of the two samples per individual was pooled to reduce stochastic variation and to facilitate the distribution of plasma over different assays. Samples were randomly assigned to plates in all assays, and all laboratory work was performed blind to any experimental groups. Immune assays were performed in the following order, to minimize the potential effect of repeated freezing and thawing for certain assays where this might negatively influence results (Hegemann et al., 2017; Liebl and Martin, 2009): (1) bacterial killing of *E. coli*; (2) hemalysis and hemagglutination; (3) haptoglobin; (4) nitric oxide; and (5) ovotransferrin.

Samples had up to five freeze–thaw cycles before the last immune test was performed, and the number of freeze–thaw cycles before a test was always consistent between samples. All assays were performed within 2 months after the last blood sample was taken except for ovotransferrin, which was performed within 12 months after sampling. Because previous experiments found no effect of storage for up to 6 years (Horrocks et al., 2011), we assumed the 12 month storage would have no effect on ovotransferrin levels.

#### Bacterial killing of *Escherichia coli*

Bacterial killing was measured using spectrophotometry, comparing bacterial growth on 96-well agar plates with and without exposure to the killing capacity of the plasma samples (Eikenaar and Hegemann, 2016; French and Neuman-Lee, 2012). Before testing our samples, we tested different plasma volumes (5 and 7 µl) and bacterial solution concentrations (10^4^ and 10^5^) to optimize the assay. In short, we mixed 7 µl of plasma with 5 µl of a 10^4^ *E. coli* solution (E power microorganisms; ACTT 8739) into each well, with additional agar instead of plasma for the positive controls. Plates were incubated at 37°C for 12 h, and were scanned hourly starting 4 h after incubation start. Scanning was done at 600 nm, using a Molecular Devices SpectraMax 340 plate reader. Samples were plated in duplicate (*r*=0.97), and each plate contained at least six positive and six negative controls. We selected the response at *t*=10 h for statistical analysis, because at that time point the response variation was largest.

#### Haptoglobin

Haptoglobin concentrations were measured using a commercial kit based on a colorimetric analysis, following the manufacturer's instructions (Tridelta development, Maynooth, Ireland), as used by Matson et al. (2012). We mixed 2.5 μl plasma with reagents in a 96-well plate, after which we recorded the absorbance at 630 nm using a Molecular Devices SpectraMax 340 plate reader.

#### Nitric oxide

Nitric oxide concentration was measured with a colorimetric assay as described by Sild and Hõrak (2009). This assay measures the concentration of both nitrate and nitrite in 10 μl of plasma. Samples were plated on 96-well plates and coloration was measured at 540 nm using a Molecular Devices SpectraMax 340 plate reader.

#### Hemolysis and hemagglutination

We measured hemolysis and hemagglutination following Matson et al. (2005). Rabbit erythrocytes (15 μl; Envigo, Huntington, UK) were incubated in serially diluted plasma. Agglutination and lysis were scored from assay plate images recorded, respectively, 20 and 90 min after incubation. Scores for both were recorded as titers. Samples were all scored twice, by the same person (M.M.G.D.), from randomized images, blind to sample ID and plate. If the two scores per sample were ≤1 titer apart, the average was used in further analysis. If these first two scores were >1 titer apart, they were scored a third time and the average of the two closest scores was used. The coefficients of variation within and between plates were 0.087 and 0.077, respectively.

#### Ovotransferrin

Ovotransferrin samples were measured as described in Horrocks et al. (2011), by estimating the amount of iron required to saturate all ovotransferrin in a 10 μl plasma sample. Samples were plated in duplicate (*r*=0.96) on 96-well plates and coloration was measured at 570 nm using a Molecular Devices SpectraMax 340 plate reader.

### Statistical analysis

Because we measured multiple immune measures per individual, we fitted multivariate response models, allowing us to estimate individual-level pairwise residual correlations between different immune measures. The models also enabled us to estimate pairwise correlations between different immune measures obtained from birds that shared parents or foster parents. Parental and plate identities were entered as Gaussian random intercepts in the models.

All analyses were conducted with R (v3.6.0; https://www.r-project.org/) in the RStudio IDE (v1.2.5019; https://www.rstudio.com/). We fitted Bayesian multivariate response models with the brms package (v2.14.0; Bürkner, 2017, 2018), interfaced with the MCMC sampler RStan (v2.21.2; https://mc-stan.org/users/interfaces/rstan).

Besides our variables of primary interest, experimental treatments, sex and age, we included season and two measures of handling stress: handling time pre-puncture and handling time post-puncture (until sample completion). We allowed for up to three-way interactions between treatment, sex and age; other predictors were entered as (additive) main effect only. For haptoglobin, we also included sample redness as additive predictor (see Table S2).

Owing to difficulties in obtaining enough blood plasma, the sample size for ovotransferrin was substantially smaller than sample sizes for the other response variables. For this reason, we fitted multivariate models with (*n*=52) and without (*n*=176) ovotransferrin.

All response variables and continuous predictors were standardized (median=0, s.d.=1) to facilitate comparison of effect sizes and to increase efficiency of the MCMC sampler. For population-level (‘fixed’) effects, we used ‘weakly informative’ (Lemoine, 2019) Gaussian priors (mean=0, s.d.=1). For group-level (‘random’) effects, we used the default priors of brms, a Student's *t* density with 3 degrees of freedom for standard deviations and an LKJ (Lewandowski et al., 2009) density for correlations.

For each model, we ran three chains with 1000 warm-up iterations, followed by 3333 sampling iterations, thus yielding almost 10,000 posterior samples per model. Proper mixing of chains was monitored with trace plots and convergence of chains by verifying that *R* values were close to 1.00 (see Tables S3 and S4). Model fits were also evaluated by inspecting posterior predictive checks, using the pp_check() function of brms.

To test hypotheses regarding model parameters, we calculated the probability of direction (*p*_d_; Makowski et al., 2019), which is the posterior probability that a quantity (parameter or derived parameter) is positive or negative, whichever is the most probable. In other words, the *p*_d_-value equals the proportion of the posterior density that has the same sign as the median of the posterior density; this value can be regarded as a Bayesian equivalent of the frequentist *P*-value. Following Makowski et al. (2019), we describe *p*_d_-values between 95% and 97% as providing ‘weak evidence’, a *p*_d_-value between 97% and 99% as ‘moderately strong evidence’ and a value greater than 99% as ‘strong evidence’.

Regarding effect sizes, we follow Cohen (1988) in considering a standardized effect size around 0.2 as ‘small’, around 0.5 as ‘medium’ and around 0.8 as ‘large’.

To calculate effect size and *p*_d_, the model posterior was fitted for the various different treatment combinations. The difference between the corresponding fits was used to test each hypothesis. In order to estimate the heritabilities of the immune indices, we fitted animal models (Kruuk, 2004). These were the same models as described above, but with an additional individual-level ‘random effect’ that corresponds to the individual's breeding value. However, because the full multivariate animal models failed to reach convergence, we only report the univariate heritabilities for each trait. In order to achieve sufficiently large effective sample sizes, we doubled the number of sampling iterations to 20,000 for the animal models.

The covariance matrix for the breeding values is the product of the additive genetic variance *V*_A_ and the relationship matrix **A**, which contains all the pairwise coefficients of relatedness between the birds in the dataset. We used the package AGHmatrix (Amadeu et al., 2016) to calculate **A** from a pedigree of the birds in our breeding colony. The packages MasterBayes (Hadfield et al., 2006) and GeneticsPed (http://www.bioconductor.org/packages/release/bioc/html/GeneticsPed.html) were used to reorder and extend the pedigree, respectively. It turned out that the birds sampled for this study comprised 394 full-sib pairs, 187 half-sib pairs, 68 cousin pairs and eight more distantly related pairs of birds; 25,678 pairs of birds were unrelated.

The univariate animal model can be written as:(1)
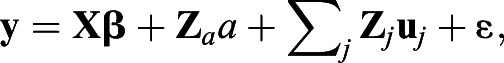
where **X** is the design matrix for the fixed effects with corresponding parameter vector **β**, **Z***_a_* is the incidence matrix for the individual breeding values *a*, the **Z***_j_* are the incidence matrices for the remaining random effects **u***_j_*, and ε are the residuals. Following de Villemereuil et al. (2018), we also included fixed effects in the heritability estimates:(2)
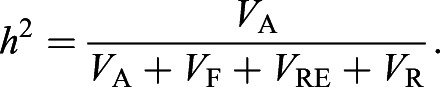


Here, *V*_F_ is variance of the estimated fixed effects, i.e. the variance of the predicted *y*-values based on fixed effect predictors, *V*_RE_ is the total variance of all remaining random effects and *V*_R_ the residual variance.

Figures were made using the ggplot2 package (Wickham, 2016). A complete list of packages used during data analysis and visualization is found in Table S5.

## RESULTS

### Developmental treatment

Average brood size after cross-fostering of the birds used in this study was 2.60 (s.d.=0.49; *n*=69 broods) and 5.5 (s.d.=0.54; *n*=37 broods) for benign and harsh developmental nests, respectively. Growth rate from age 1–5 days to age 14–16 days was 0.13 g day^–1^ (range 0.10–0.17 g day^–1^) lower in the large broods compared with the small broods (*p*_d_=1). To put this into context, the average growth rate was 0.73 g day^–1^ (s.d.=0.14 g day^–1^), and the average full-grown finch in this experiment weighed 14.4 g (s.d.=1.55 g). This difference in growth rate led to a difference of 1.4 g at fledging age (*p*_d_=1), and translated into a smaller but significant difference of 0.7 g when fully grown (*p*_d_=1). These results are consistent with effects observed previously by Briga et al. (2017).

### Immune function

We tested six innate immune components in plasma of 230 zebra finches. Only three birds showed lysis and only 13 showed *E. coli* killing capacity, hence we omitted these two immune parameters from further analysis. We also omitted three samples for all immune parameters, because of extremely high haptoglobin and/or nitric oxide values (>5 standard deviations above the mean). Such extremely high values are indicative of infection, inflammation or trauma, reducing the sample size to 227.

For various samples, plasma shortage or failed tests led to more samples being taken out for multivariate analysis, and we ended up with 176 and 52 complete samples for the two statistical models. Table 1 shows the distribution of treatment and sex within the datasets.

**Table 1.**
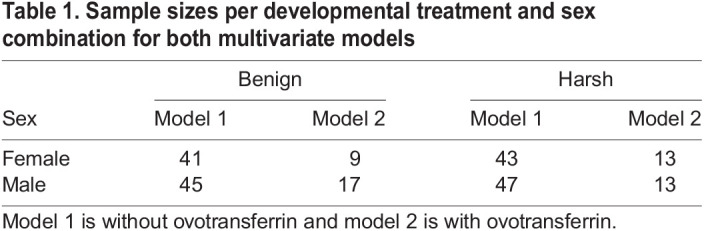
Sample sizes per developmental treatment and sex combination for both multivariate models

#### Developmental treatment

There was no clear evidence for effects of developmental treatment on haptoglobin, nitric oxide, agglutination and ovotransferrin, with probability of direction for all effects below 0.95. There was also no evidence for sex- or age-specific effects of the treatment (Table 2).

**Table 2.**
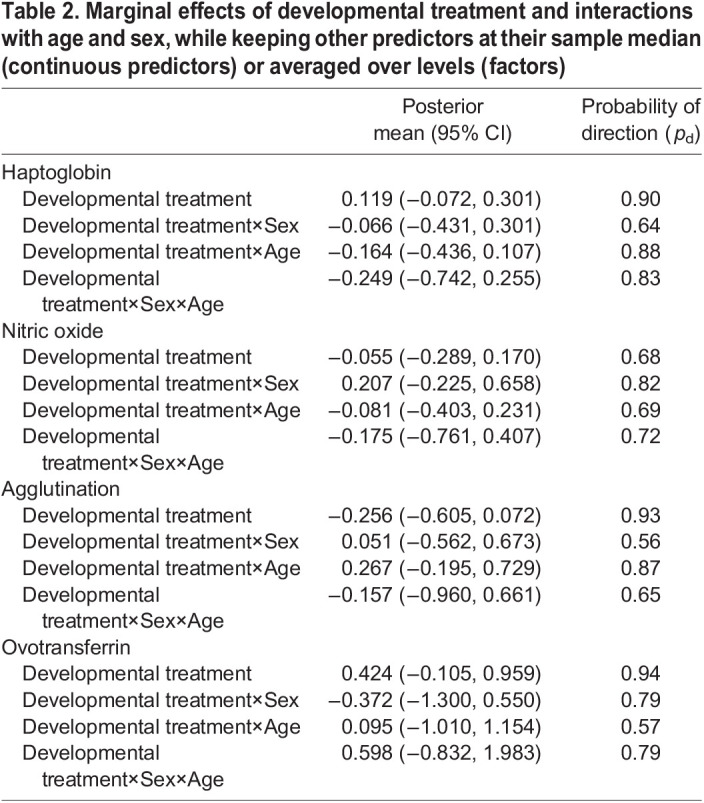
Marginal effects of developmental treatment and interactions with age and sex, while keeping other predictors at their sample median (continuous predictors) or averaged over levels (factors)

#### Sex and age

**Fig. 1.**
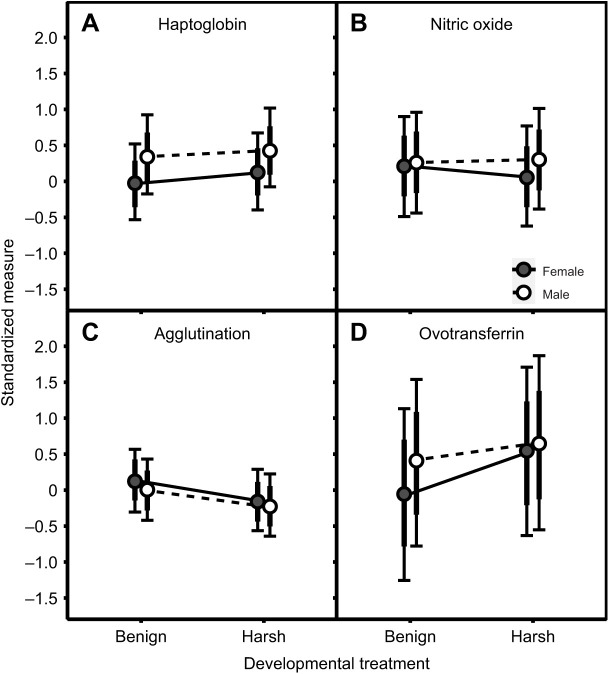
**Sex-specific effects of developmental treatment on four immune indices in zebra finches.** (A) Haptoglobin; (B) nitric oxide; (C) agglutination; (D) ovotransferrin. Data are shown as posterior means (circles) and 80% (thick bars) and 95% credible intervals (thin bars with whiskers) for males (white, dashed line) and females (gray, solid line). On the *y*-axis, we show the standardized concentration (A,B,D) or standardized titre (C). *N*=176 for A–C, and *N*=52 for D.

**Fig. 2.**
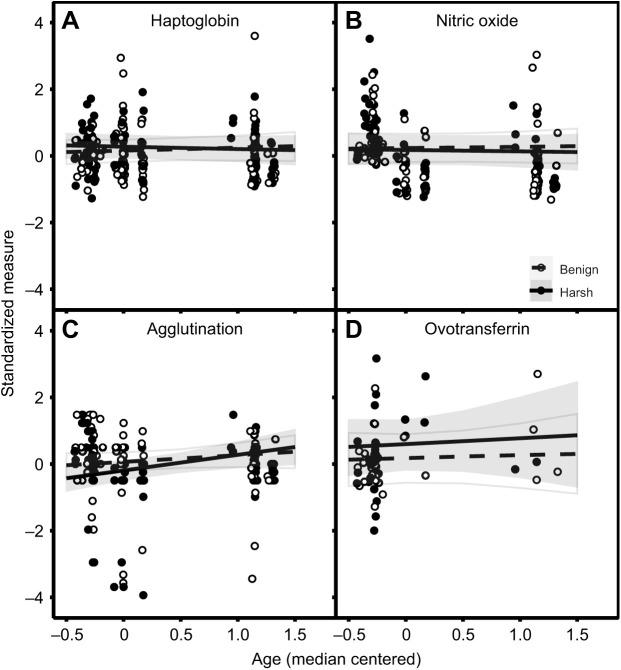
**Age-specific effects of benign (white, dashed line) and harsh (black, solid line) developmental treatment on four immune indices in zebra finches.** (A) Haptoglobin; (B) nitric oxide; (C) agglutination; (D) ovotransferrin. Immune values are median centered on the *y*-axis, and age is median centered on the *x*-axis. On the *y*-axis, we show the standardized concentration (A,B,D) or standardized titre (C). Lines represent the mean effects as predicted by the model, with shaded areas representing 95% credible intervals and standardized data points shown as circles. *N*=176 for A–C, and *N*=52 for D.

Males, on average, had higher values for haptoglobin (small effect: 0.34; *p*_d_=1.00) than females, while there was no evidence for a sex effect on agglutination, nitric oxide and ovotransferrin (Fig. 1; Table S6). Agglutination levels increased with age (small effect: 0.33; *p*_d_=0.96), whilst haptoglobin, nitric oxide and ovotransferrin were all independent of age, as indicated by probability of direction estimates below 0.95 (Fig. 2; Table S6).

#### Correlations between immune measures

Haptoglobin and nitric oxide residuals were positively correlated (*r*=0.178, *p*_d_=0.97) when accounting for all fixed and random effects (Table 3), while there was a negative correlation between agglutination and ovotransferrin (*r*=−0.347, *p*_d_=0.98). Other correlations among immune measures were weaker, with probability of direction below 0.95.

**Table 3.**
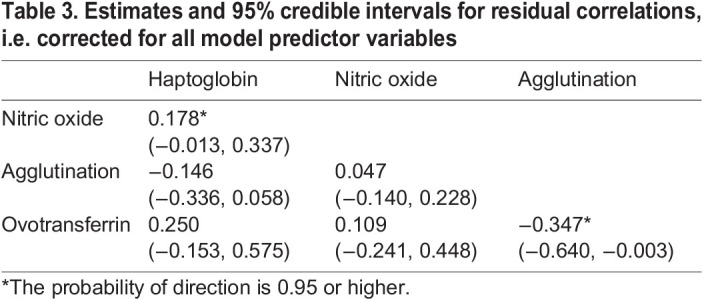
Estimates and 95% credible intervals for residual correlations, i.e. corrected for all model predictor variables

#### Heritabilities

Heritabilities of all immune measures were relatively small, with *h*^2^ point estimates ranging from 0.044 to 0.107, and upper limits of credible intervals up to 0.389 (Table 4).

**Table 4.**
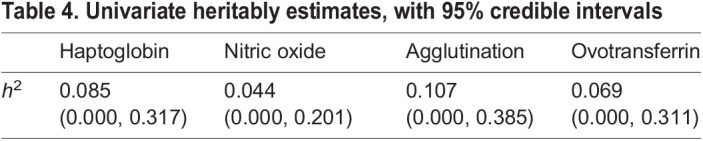
Univariate heritably estimates, with 95% credible intervals

## DISCUSSION

We tested whether birds that grew up in large broods displayed a decreased immune function as adults. However, we found no clear evidence for any effects of early developmental condition on innate immune function in adulthood. These results are in line with some of the scarce results in the literature on brood size manipulation effects and other manipulations of early-life conditions, with no observed negative effects of developmental hardship on immune function during adulthood (Kriengwatana et al., 2013; Schmidt et al., 2015; Tschirren et al., 2009). However, results from Kriengwatana et al. (2013) show that effects of developmental treatments may vary depending on circumstances later in life; they found that effects of food manipulation during early development on part of the immune system varied with food availability during the later juvenile stage. Therefore, we speculate that effects of developmental hardship on the immune system may be exacerbated had the birds experienced a more challenging environment in adulthood. Indeed, Briga et al. (2017) reported the effect of developmental conditions on lifespan to be strongest in harsh foraging conditions, where it was more energy costly for animals to obtain food.

The immune system is complex and it remains an open question as to how many immune indices need to be measured to yield a sufficiently comprehensive description of immune function (assuming that this is possible at all). Correlations between indices are informative in this respect, with low correlations indicating that more indices are needed compared with high correlations. We found that haptoglobin and nitric oxide showed a weak positive correlation, while agglutination and ovotransferrin showed a weak negative correlation. No evidence for further correlations amongst measured immune indices was found. These findings underline the importance of measuring a multitude of different immune components when investigating immune function, in line with earlier studies that reached the same conclusion (Matson et al., 2006; Roast et al., 2019). High values in one immune component do not automatically predict that the individual is immunocompetent overall. There might very well be trade-offs and differential investment between the various components, depending on, for instance, environment, life history and species. This also holds for observed effects of treatments, as evident from our results. Previously discussed results from Kriengwatana et al. (2013) also show that environmental variables such as food availability can influence one immune variable whilst another stays unaffected. Therefore, great care needs to be taken when making predictions based on a single component or part of the immune system.

Phenotypic variation can be attributed to a combination of environmental and genetic effects. Given that an important environmental component, brood size, had negligible effects on innate immune function, this raised the question as to what extent additive genetic effects explained individual variation in innate immune function. Using animal models, and making use of an extensive pedigree, we estimated that additive genetic effects explained 4–11% of the variation in the different immune indices. This suggests that the evolutionary benefit of highly heritable immune traits is limited, potentially owing to changes in pathogenic pressure between generations. There is limited literature for comparison, but these values are in the range of what would be expected for populations living in a variable environment, where phenotypic plasticity can play a dominant role. Looking at repeatability of traits can provide an upper bound to heritability estimates (Greives et al., 2017), and repeatability of immune measures in semi-wild settings is generally low, with most repeatability estimates below 0.3 (e.g. Buehler et al., 2008b; Nwaogu et al., 2019; Roast et al., 2019). The semi-controlled conditions within our setup remove much environmental variation, but still expose the animals to natural variation in weather and presumably pathogen pressure, which might be enough to result in the observed low heritability. Even with *ad libitum* food and semi-sheltered conditions, there is high variation in these measures of innate immunity. This can be interpreted as highlighting the importance of environmental variation when studying the immune system, a narrative that is gaining more ground within the field of ecological immunology (Tieleman, 2018). But given that the environmental variation for our populations is the same for all individuals, the cause of the observed inter-individual variation in innate immune function has to lie elsewhere. Alternatively, complexity of the immune system, in particular the fact that there can be different ways to achieve resistance, is such that dynamics within the system can generate very different outcomes given a starting point determined by environment and genetic make-up (Das et al., 2020), at least when looking at lower-level immune indices as opposed to organism-level performance in resisting a specific pathogen.

## Supplementary Material

Supplementary information
