# Supplementary material for: Effects of early-life conditions on innate immune function in adult zebra finches
Source: J Exp Biol. 2021 Jun 4;224(11):jeb242158. doi: 10.1242/jeb.242158 (PMC8214827; doi:10.1242/jeb.242158)
Supplement: Supplementary information [file jexbio-224-242158-s1.pdf]

Table S1: descriptions of the immune system aspects measured per immune assay.

**Measure**	**Description**
E. coli killing	The Bacterial killing assay (BKA) measures the capacity of fresh blood or plasma to kill micro-organisms of specific species or strains ex-vivo. The assay measures the functional immune response against the pathogens, involving several components of the innate immune system, dependent on the species and strain of the micro-organism. In this specific case with *E. coli*, defense is complement-dependent. Increased killing capacity is considered indicative of a stronger immune response.
Hemagglutination	Hemagglutination is a measure of natural antibodies (NAbs). This is a special group of immunoglobulin molecules, which are part of the innate immune system and are not dependent on previous exposure to a particular antigen. Most NABs are pentameric IgM, but some dimeric (IgA) and monomeric (IgG/IgY) have been reported (Avrameas, 1991).
Hemolysis	Hemolysis is a measure of complement activity. The complement system is a complex enzyme cascade that leads to cell lysis. Higher baseline levels are interpreted as better immune function.
Haptoglobin	Haptoglobin (Hp) is a positive acute phase protein that binds hemoglobin. It is normally present at low levels in the blood stream, but can drastically increase in response to acute infection or inflammation. Haptoglobin also has antimicrobial effects. Baseline levels are the best known predictor of response Haptoglobin levels, with higher baseline levels correlating with an increased ability to mount an haptoglobin response to an immune challenge (Matson et al., 2012).
Nitric Oxide	Nitric oxide (NOx) is a signaling molecule that is produced by macrophages. It can modulate inflammatory processes and is toxic to bacteria and intracellular parasites (Seguin et al., 1994). Higher baseline levels thought to contribute to improved immune function.
Ovotransferrin	Ovotransferrin is a positive acute-phase protein in birds, with iron binding and immunomodulatory functions. Its capability to bind free iron, an essential nutrient for bacterial growth (Skaar, 2010), limits infection by both gram-positive and gram-negative bacteria (Superti et al., 2007; Valenti et al., 1983). Ovotransferrin also has a bactericidal domain (Ibrahim et al., 1998), and exhibits further antifungal (Valenti et al., 1985) and antiviral (Giansanti et al., 2002; Giansanti et al., 2007) bioactivities. Higher baseline levels are interpreted as being beneficial for immune function.

Journal of Experimental Biology • Supplementary information

Table S2: Model formulas for each of the response variables. Predictor variables are split into population-level (fixed) effects and group-level (random) effects.

Response variable	Population level effects formula	Group level effects formula
Haptoglobin	~ DT*Sex*Age + Season + Handling time pre puncture + Handling time post puncture + Sample redness	(1|Plate) + (1|Mother) + (1|Father)
Nitric oxide	~ DT*Sex*Age + Season + Handling time pre puncture + Handling time post puncture	(1|Plate) + (1|Mother) + (1|Father)
Agglutination	~ DT*Sex*Age + Season + Handling time pre puncture + Handling time post puncture	(1|Mother) + (1|Father)
Ovotransferrin	~ DT*Sex*Age + Season + Handling time pre puncture + Handling time post puncture	(1|Plate) + (1|Mother) + (1|Father)

Table S3: Model output, including estimates (Estimate; posterior mean), estimation error (Est.Error; posterior standard deviation), effective samples sizes (Bulk ESS, Tail ESS) and Rhat values for model 1, without ovotransferrin. Number of observations: 176.

**Group-Level Effects:**							
~FemaleID	Number of levels: 61						
	Estimate	Est.Error	l-95% CI	u-95% CI	Bulk ESS	Tail ESS	Rhat
sd(HpStd_Intercept)	0.16	0.11	0.01	0.39	1110	2462	1.00
sd(NOxStd_Intercept)	0.13	0.09	0.01	0.33	2252	4350	1.00
sd(HAStd_Intercept)	0.26	0.15	0.01	0.56	1504	2335	1.00
cor(HpStd_Intercept,NOxStd_Intercept)	0.04	0.49	-0.87	0.88	4656	6278	1.00
cor(HpStd_Intercept,HAStd_Intercept)	0.01	0.48	-0.84	0.87	3144	4805	1.00
cor(NOxStd_Intercept,HAStd_Intercept)	0.02	0.49	-0.86	0.87	2738	4884	1.00
~MaleID	Number of levels: 62						
	Estimate	Est.Error	l-95% CI	u-95% CI	Bulk ESS	Tail ESS	Rhat
sd(HpStd_Intercept)	0.27	0.12	0.03	0.48	915	1371	1.00
sd(NOxStd_Intercept)	0.15	0.10	0.01	0.36	1615	3015	1.00
sd(HAStd_Intercept)	0.26	0.16	0.01	0.58	1500	3430	1.00
cor(HpStd_Intercept,NOxStd_Intercept)	-0.06	0.47	-0.88	0.83	3768	5461	1.00
cor(HpStd_Intercept,HAStd_Intercept)	-0.10	0.46	-0.88	0.81	3309	3892	1.00
cor(NOxStd_Intercept,HAStd_Intercept)	0.07	0.49	-0.85	0.90	2762	5056	1.00
~Round:Hp_Plate	Number of levels: 9						
	Estimate	Est.Error	l-95% CI	u-95% CI	Bulk ESS	Tail ESS	Rhat
sd(HpStd_Intercept)	0.44	0.21	0.14	0.97	2007	2735	1.00
~Round:NOx_Plate	Number of levels: 8						
	Estimate	Est.Error	l-95% CI	u-95% CI	Bulk ESS	Tail ESS	Rhat
sd(NOxStd_Intercept)	0.61	0.28	0.25	1.32	2983	4147	1.00
**Population-Level Effects:**							
	Estimate	Est.Error	l-95% CI	u-95% CI	Bulk ESS	Tail ESS	Rhat
HpStd_Intercept	0.00	0.26	-0.47	0.54	3435	4327	1.00
NOxStd_Intercept	-0.04	0.36	-0.71	0.74	3458	4180	1.00
HAStd_Intercept	-0.37	0.21	-0.78	0.06	7230	7696	1.00
HpStd_DTharsh	0.15	0.14	-0.12	0.42	4552	6133	1.00
HpStd_Sex2	0.37	0.15	0.08	0.65	3993	6012	1.00
HpStd_Age_Mc	-0.01	0.17	-0.34	0.31	4575	6217	1.00
HpStd_Redness_Std	0.06	0.08	-0.11	0.22	5331	7168	1.00
HpStd_AvgHandlingTime_Pre_Std	0.02	0.05	-0.08	0.12	8666	7751	1.00
HpStd_AvgHandlingTime_Post_Std	0.21	0.05	0.10	0.31	9123	7998	1.00
HpStd_Season2	-0.04	0.34	-0.72	0.63	4229	4661	1.00

Journal of Experimental Biology • Supplementary information

HpStd_DTharsh:Sex2	-0.07	0.19	-0.43	0.31	3752	6231	1.00
HpStd_DTharsh:Age_Mc	-0.04	0.20	-0.43	0.34	4735	5935	1.00
HpStd_Sex2:Age_Mc	0.21	0.20	-0.18	0.59	4165	5555	1.00
HpStd_DTharsh:Sex2:Age_Mc	-0.25	0.26	-0.75	0.25	4541	7045	1.00
NOxStd_DTharsh	-0.16	0.16	-0.48	0.16	5423	7514	1.00
NOxStd_Sex2	0.05	0.17	-0.28	0.36	5357	7489	1.00
NOxStd_Age_Mc	-0.05	0.18	-0.41	0.31	6165	6935	1.00
NOxStd_AvgHandlingTime_Pre_Std	-0.01	0.06	-0.13	0.11	10495	6544	1.00
NOxStd_AvgHandlingTime_Post_Std	0.06	0.06	-0.07	0.18	10420	7512	1.00
NOxStd_Season2	0.51	0.45	-0.45	1.32	4312	5476	1.00
NOxStd_DTharsh:Sex2	0.21	0.22	-0.23	0.66	4882	6856	1.00
NOxStd_DTharsh:Age_Mc	-0.10	0.23	-0.54	0.34	5405	7318	1.00
NOxStd_Sex2:Age_Mc	0.17	0.22	-0.26	0.59	5205	7296	1.00
NOxStd_DTharsh:Sex2:Age_Mc	0.03	0.30	-0.54	0.62	5190	6913	1.00
HAStd_DTharsh	-0.28	0.23	-0.74	0.18	5791	7358	1.00
HAStd_Sex2	-0.12	0.24	-0.58	0.34	5300	7357	1.00
HAStd_Age_Mc	0.04	0.26	-0.47	0.56	5430	6606	1.00
HAStd_AvgHandlingTime_Pre_Std	0.03	0.09	-0.15	0.20	10051	7328	1.00
HAStd_AvgHandlingTime_Post_Std	0.05	0.09	-0.14	0.23	10373	7755	1.00
HAStd_Season2	0.98	0.25	0.48	1.47	7769	7189	1.00
HAStd_DTharsh:Sex2	0.05	0.31	-0.56	0.67	4791	6531	1.00
HAStd_DTharsh:Age_Mc	0.27	0.32	-0.35	0.90	5070	6211	1.00
HAStd_Sex2:Age_Mc	0.32	0.31	-0.28	0.94	4850	6717	1.00
HAStd_DTharsh:Sex2:Age_Mc	-0.01	0.41	-0.82	0.77	4576	6693	1.00
**Family Specific Parameters:**							
	Estimate	Est.Error	l-95% CI	u-95% CI	Bulk ESS	Tail ESS	Rhat
sigma_HpStd	0.52	0.04	0.44	0.61	2088	4002	1.00
sigma_NOxStd	0.66	0.04	0.58	0.75	5480	7713	1.00
sigma_HAStd	0.98	0.07	0.86	1.12	4545	6546	1.00
**Residual Correlations:**							
	Estimate	Est.Error	l-95% CI	u-95% CI	Bulk ESS	Tail ESS	Rhat
rescor(HpStd,NOxStd)	0.18	0.09	-0.01	0.36	6712	6910	1.00
rescor(HpStd,HAStd)	-0.15	0.1	-0.34	0.06	5709	6353	1.00
rescor(NOxStd,HAStd)	0.05	0.09	-0.14	0.23	8002	7393	1.00

Journal of Experimental Biology • Supplementary information

Table S4: Model output, including estimates (Estimate; posterior mean), estimation error (Est.Error; posterior standard deviation), effective samples sizes (Bulk ESS, Tail ESS) and Rhat values for model 2, including ovotransferrin. Number of observations: 52.

**Group-Level Effects:**							
~FemaleID	Number of levels: 28						
	Estimate	Est.Error	l-95% CI	u-95% CI	Bulk ESS	Tail ESS	Rhat
sd(HpStd_Intercept)	0.14	0.10	0.01	0.39	2843	4482	1.00
sd(NOxStd_Intercept)	0.11	0.08	0.00	0.30	3874	4400	1.00
sd(HAStd_Intercept)	0.32	0.22	0.01	0.80	2388	2875	1.00
sd(OvoStd_Intercept)	0.23	0.17	0.01	0.64	3596	4498	1.00
cor(HpStd_Intercept,NOxStd_Intercept)	0.00	0.45	-0.81	0.81	7669	6910	1.00
cor(HpStd_Intercept,HAStd_Intercept)	-0.06	0.44	-0.83	0.78	5103	6489	1.00
cor(NOxStd_Intercept,HAStd_Intercept)	-0.02	0.45	-0.83	0.80	5520	6783	1.00
cor(HpStd_Intercept,OvoStd_Intercept)	0.11	0.45	-0.77	0.85	6526	6481	1.00
cor(NOxStd_Intercept,OvoStd_Intercept)	-0.02	0.45	-0.82	0.80	7240	7964	1.00
cor(HAStd_Intercept,OvoStd_Intercept)	-0.01	0.45	-0.82	0.82	7232	8106	1.00
~MaleID	Number of levels: 30						
	Estimate	Est.Error	l-95% CI	u-95% CI	Bulk ESS	Tail ESS	Rhat
sd(HpStd_Intercept)	0.16	0.12	0.01	0.43	1906	3940	1.00
sd(NOxStd_Intercept)	0.14	0.10	0.01	0.37	2790	3900	1.00
sd(HAStd_Intercept)	0.45	0.26	0.02	0.99	1786	3011	1.00
sd(OvoStd_Intercept)	0.23	0.17	0.01	0.63	3081	3692	1.00
cor(HpStd_Intercept,NOxStd_Intercept)	-0.07	0.44	-0.84	0.78	5394	5654	1.00
cor(HpStd_Intercept,HAStd_Intercept)	-0.16	0.44	-0.87	0.73	2937	5456	1.00
cor(NOxStd_Intercept,HAStd_Intercept)	0.12	0.44	-0.77	0.84	3626	5974	1.00
cor(HpStd_Intercept,OvoStd_Intercept)	0.11	0.45	-0.78	0.86	6327	7253	1.00
cor(NOxStd_Intercept,OvoStd_Intercept)	0.00	0.45	-0.80	0.82	6453	6953	1.00
cor(HAStd_Intercept,OvoStd_Intercept)	0.02	0.44	-0.79	0.82	6578	7747	1.00
~Round:Hp_Plate	Number of levels: 7						
	Estimate	Est.Error	l-95% CI	u-95% CI	Bulk ESS	Tail ESS	Rhat
sd(HpStd_Intercept)	0.46	0.41	0.02	1.45	1592	2620	1.00
~Round:NOx_Plate	Number of levels: 5						
	Estimate	Est.Error	l-95% CI	u-95% CI	Bulk ESS	Tail ESS	Rhat
sd(NOxStd_Intercept)	0.42	0.47	0.01	1.65	2013	3835	1.00
~Round:Ovo_Plate	Number of levels: 8						
	Estimate	Est.Error	l-95% CI	u-95% CI	Bulk ESS	Tail ESS	Rhat
sd(OvoStd_Intercept)	1.05	0.49	0.36	2.23	2736	3572	1.00
**Population-Level Effects:**							

Journal of Experimental Biology • Supplementary information

	Estimate	Est.Error	l-95% CI	u-95% CI	Bulk ESS	Tail ESS	Rhat
HpStd_Intercept	-0.44	0.40	-1.19	0.33	4720	4119	1.00
NOxStd_Intercept	-0.29	0.43	-1.08	0.65	4466	4090	1.00
HAStd_Intercept	-0.70	0.49	-1.65	0.25	6811	7034	1.00
OvoStd_Intercept	0.09	0.65	-1.20	1.35	5001	6291	1.00
HpStd_DTharsh	0.26	0.24	-0.22	0.74	3969	5802	1.00
HpStd_Sex2	0.47	0.22	0.04	0.91	4076	6352	1.00
HpStd_Age_Mc	0.09	0.32	-0.53	0.72	4669	6629	1.00
HpStd_Redness_Std	-0.30	0.28	-0.82	0.29	2801	4251	1.00
HpStd_AvgHandlingTime_Pre_Std	-0.11	0.11	-0.32	0.11	6318	6432	1.00
HpStd_AvgHandlingTime_Post_Std	0.30	0.12	0.05	0.55	6625	6631	1.00
HpStd_Season2	0.03	0.48	-0.86	1.05	3681	4727	1.00
HpStd_DTharsh:Sex2	-0.47	0.29	-1.05	0.11	4572	6567	1.00
HpStd_DTharsh:Age_Mc	0.59	0.47	-0.36	1.47	3981	5522	1.00
HpStd_Sex2:Age_Mc	-0.11	0.36	-0.81	0.58	4669	6846	1.00
HpStd_DTharsh:Sex2:Age_Mc	-0.43	0.55	-1.48	0.67	5395	6564	1.00
NOxStd_DTharsh	-0.02	0.22	-0.46	0.42	5034	6866	1.00
NOxStd_Sex2	0.19	0.22	-0.24	0.61	4324	6124	1.00
NOxStd_Age_Mc	-0.28	0.30	-0.87	0.31	5490	6376	1.00
NOxStd_AvgHandlingTime_Pre_Std	-0.03	0.11	-0.26	0.19	6096	7116	1.00
NOxStd_AvgHandlingTime_Post_Std	-0.16	0.12	-0.40	0.09	6665	7387	1.00
NOxStd_Season2	0.32	0.47	-0.71	1.18	4512	4433	1.00
NOxStd_DTharsh:Sex2	-0.01	0.29	-0.57	0.55	4654	6008	1.00
NOxStd_DTharsh:Age_Mc	0.29	0.44	-0.58	1.14	5357	6907	1.00
NOxStd_Sex2:Age_Mc	0.32	0.33	-0.34	0.97	5885	6760	1.00
NOxStd_DTharsh:Sex2:Age_Mc	-0.48	0.54	-1.51	0.60	5243	6726	1.00
HAStd_DTharsh	0.30	0.42	-0.51	1.12	5438	6225	1.00
HAStd_Sex2	-0.01	0.39	-0.79	0.76	5676	6866	1.00
HAStd_Age_Mc	0.40	0.53	-0.65	1.45	6110	6630	1.00
HAStd_AvgHandlingTime_Pre_Std	-0.11	0.23	-0.55	0.34	6989	7372	1.00
HAStd_AvgHandlingTime_Post_Std	0.05	0.26	-0.44	0.56	6318	7076	1.00
HAStd_Season2	0.72	0.52	-0.32	1.73	7170	7206	1.00
HAStd_DTharsh:Sex2	-0.40	0.52	-1.42	0.62	5058	7256	1.00
HAStd_DTharsh:Age_Mc	0.19	0.65	-1.10	1.48	9858	7958	1.00
HAStd_Sex2:Age_Mc	0.15	0.57	-0.96	1.26	7670	7786	1.00
HAStd_DTharsh:Sex2:Age_Mc	0.04	0.75	-1.45	1.54	8457	8076	1.00
OvoStd_DTharsh	0.61	0.37	-0.11	1.33	4493	5795	1.00
OvoStd_Sex2	0.46	0.35	-0.23	1.14	4861	6193	1.00
OvoStd_Age_Mc	0.11	0.48	-0.80	1.05	6317	7251	1.00
OvoStd_AvgHandlingTime_Pre_Std	0.11	0.20	-0.29	0.50	6712	6914	1.00
OvoStd_AvgHandlingTime_Post_Std	0.36	0.23	-0.08	0.80	7434	7338	1.00
OvoStd_Season2	-0.30	0.66	-1.57	1.01	5971	7022	1.00
OvoStd_DTharsh:Sex2	-0.37	0.47	-1.29	0.57	4553	5500	1.00
OvoStd_DTharsh:Age_Mc	0.13	0.62	-1.11	1.33	6837	7085	1.00

OvoStd_Sex2:Age_Mc	-0.06	0.51	-1.09	0.96	7219	7103	1.00
OvoStd_DTharsh:Sex2:Age_Mc	-0.08	0.72	-1.47	1.33	7459	7426	1.00
**Family Specific Parameters:**							
	Estimate	Est.Error	l-95% CI	u-95% CI	Bulk ESS	Tail ESS	Rhat
sigma_HpStd	0.45	0.07	0.32	0.60	2925	4585	1.00
sigma_NOxStd	0.47	0.06	0.36	0.61	4859	6331	1.00
sigma_HAStd	1.02	0.15	0.76	1.33	3541	5664	1.00
sigma_OvoStd	0.86	0.12	0.66	1.11	5104	7152	1.00
**Residual Correlations:**							
	Estimate	Est.Error	l-95% CI	u-95% CI	Bulk ESS	Tail ESS	Rhat
rescor(HpStd,NOxStd)	0.08	0.20	-0.32	0.47	3900	5813	1.00
rescor(HpStd,HAStd)	-0.02	0.20	-0.40	0.38	3409	4894	1.00
rescor(NOxStd,HAStd)	0.20	0.18	-0.16	0.52	5269	7065	1.00
rescor(HpStd,OvoStd)	0.25	0.19	-0.15	0.58	4614	6433	1.00
rescor(NOxStd,OvoStd)	0.11	0.18	-0.24	0.45	5430	6299	1.00
rescor(HAStd,OvoStd)	-0.35	0.16	-0.64	0.00	5463	6929	1.00

Journal of Experimental Biology • Supplementary information

Table S5: A list of used packages in R during data analysis and visualization.

Package	Version	Reference
AGHmatrix	2.0.0	(Amadeu et al., 2016)
Bayesplot	1.7.2	(Gabry and Mahr, 2020)
Brms	2.14.4	(Bürkner, 2017; Bürkner, 2018)
Car	3.0.10	(Fox and Weisberg, 2019)
Cowplot	1.1.0	(Wilke, 2019)
Dplyr	1.0.2	(Wickham et al., 2020)
GeneticsPed	1.52.0	(Gorjanc et al., 2020)
ggplot2	3.3.2	(Wickham, 2016)
ggpubr	0.4.0	(Kassambara, 2018)
ggthemes	4.2.0	(Arnold, 2019)
loo	2.3.1	(Vehtari et al., 2019)
MasterBayes	2.57	(Hadfield et al., 2006)
MCMCglmm	2.29	(Hadfield, 2010)
Mice	3.11.0	(Buuren and Groothuis-Oudshoorn, 2011)
Officer	0.3.15	(Gohel, 2019)
RODBC	1.3.17	(Ripley and Lapsley, 2017)
Rstan	2.21.2	(Stan Development Team, 2020)
Tidybayes	2.3.1	(Kay, 2020)

Journal of Experimental Biology • Supplementary information

Table S6: Additional effects of Sex and Age. The table shows the estimates and 95% credible intervals, as well as the probability of direction, for all immune measure

**a) Haptoglobin**	Posterior mean (95% CI)	Probability of direction (*pd*)	**c) Agglutination**	Posterior mean (95% CI)	Probability of direction (*pd*)
Sex	0.336 (0.123, 0.533)	1.00	Sex	-0.092 (-0.421, 0.263)	0.70
Age	0.009 (-0.201, 0.23)	0.53	Age	0.334 (-0.03, 0.692)	0.96
Sex x Age	0.081 (-0.179, 0.364)	0.72	Sex x Age	0.313 (-0.162, 0.759)	0.91
**b) Nitric Oxide**			**d) Ovotransferrin**		
Sex	0.149 (-0.073, 0.393)	0.90	Sex	0.274 (-0.276, 0.833)	0.84
Age	-0.002 (-0.243, 0.253)	0.50	Age	0.129 (-0.713, 0.948)	0.62
Sex x Age	0.187 (-0.139, 0.505)	0.87	Sex x Age	-0.101 (-1.171, 0.918)	0.58

Dataset 1

Click here to download Dataset 1

Journal of Experimental Biology • Supplementary information

References

Amadeu, R. R., Cellon, C., Olmstead, J. W., Garcia, A. A. F., Resende, M. F. R. and Muñoz, P. R. (2016). AGHmatrix: R Package to Construct Relationship Matrices for Autotetraploid and Diploid Species: A Blueberry Example. *Plant Genome* **9**,.

Arnold, J. B. (2019). ggthemes: Extra Themes, Scales and Geoms for “ggplot2.”

Avrameas, S. (1991). Natural autoantibodies: from “horror autotoxicus” to “gnothi seauton.” *Immunol. Today* **12**, 154–159.

Bürkner, P. C. (2017). brms: An R package for Bayesian multilevel models using Stan. *J. Stat. Softw.* **80**,.

Bürkner, P. C. (2018). Advanced Bayesian multilevel modeling with the R package brms. *R J.* **10**, 395–411.

Buuren, S. van and Groothuis-Oudshoorn, K. (2011). mice: Multivariate Imputation by Chained Equations in R. *J. Stat. Softw.* **45**, 1–67.

Fox, J. and Weisberg, S. (2019). *An R Companion to Applied Regression*. Third edit. Thousand Oaks CA: Sage.

Gabry, J. and Mahr, T. (2020). bayesplot: Plotting for Bayesian Models.

Giansanti, F., Rossi, P., Massucci, M., Botti, D., Antonini, G., Valenti, P. and Seganti, L. (2002). Antiviral activity of ovotransferrin discloses an evolutionary strategy for the defensive activities of lactoferrin. *Biochem. Cell Biol.* **80**, 125–130.

Giansanti, F., Giardi, M., Massucci, M., Botti, D. and Antonini, G. (2007). Ovotransferrin expression and release by chicken cell lines infected with Marek’s disease virus. *Biochem. Cell Biol.* **85**, 150–155.

Gohel, D. (2019). officer: Manipulation of Microsoft Word and PowerPoint Documents.

Gorjanc, G., Henderson, D. A., Kinghorn, with code contributions by B. and Andrew, P. (2020). GeneticsPed: Pedigree and genetic relationship functions.

Hadfield, J. D. (2010). MCMC Methods for Multi-Response Generalized Linear Mixed Models: The MCMCglmm R Package. *J. Stat. Softw.* **33**, 1–22.

Hadfield, J. D., Richardson, D. S. and Burke, T. (2006). Towards unbiased parentage assignment: combining genetic, behavioural and spatial data in a Bayesian framework. *Mol. Ecol.* **15**,.

Ibrahim, H. R., Iwamori, E., Sugimoto, Y. and Aoki, T. (1998). Identification of a distinct antibacterial domain within the N-lobe of ovotransferrin. *Biochim. Biophys. Acta - Mol. Cell Res.* **1401**, 289–303.

Kassambara, A. (2018). ggpubr: “ggplot2” Based Publication Ready Plots.

Kay, M. (2020). tidybayes: Tidy Data and Geoms for Bayesian Models.

Matson, K. D., Horrocks, N. P. C., Versteegh, M. A. and Tieleman, B. I. (2012). Baseline haptoglobin concentrations are repeatable and predictive of certain aspects of a subsequent experimentally-induced inflammatory response. *Comp. Biochem. Physiol. - A Mol. Integr. Physiol.* **162**, 7–15.

Ripley, B. and Lapsley, M. (2017). RODBC: ODBC Database Access.

Seguin, B. M. C., Klotz, F. W., Schneider, I., Weir, J. P., Goodbary, M., Slayter, M., Raney, J. J., Aniagolu, J. U. and Green, S. J. (1994). Induction of Nitric Oxide Synthase Protects against

Malaria in Mice Exposed to Irradiated Plasmodium berghei Infected Mosquitoes: Involvement of Interferon y and CD8 + T Cells. *J. Exp. Med.* **180**, 4–6.

Skaar, E. P. (2010). The Battle for Iron between Bacterial Pathogens and Their Vertebrate Hosts. *PLOS Pathog.* **6**, e1000949.

Stan Development Team (2020). RStan: the R interface to Stan.

Superti, F., Ammendolia, M. G., Berlutti, F. and Valenti, P. (2007). Ovotransferrin. In *Bioactive Egg Compounds* (ed. Huopalahti, R.), López-Fandiño, R.), Anton, M.), and Schade, R.), pp. 43–50. Berlin &Heidelberg: Springer-Verlag.

Valenti, P., Antonini, G., Von Hunolstein, C., Visca, P., Orsi, N. and Antonini, E. (1983). Studies of the antimicrobial activity of ovotransferrin. *Int. J. Tissue React.* **5**, 97—105.

Valenti, P., Visca, P., Antonini, G. and Orsi, N. (1985). Antifungal activity of ovotransferrin towards genus Candida. *Mycopathologia* **89**, 169—175.

Vehtari, A., Gabry, J., Yao, Y. and Gelman, A. (2019). loo: Efficient leave-one-out cross-validation and WAIC for Bayesian models.

Wickham, H. (2016). *ggplot2: Elegant Graphics for Data Analysis.* New York: Springer-Verlag.

Wickham, H., François, R., Henry, L. and Müller, K. (2020). dplyr: A Grammar of Data Manipulation.

Wilke, C. O. (2019). cowplot: Streamlined Plot Theme and Plot Annotations for “ggplot2.”
